# Editorial: Plant cell factories: current and future uses of plant cell cultures

**DOI:** 10.3389/fpls.2024.1439261

**Published:** 2024-06-05

**Authors:** Suvi Tuulikki Häkkinen, Sylvain Legay, Heiko Rischer, Jenny Renaut, Gea Guerriero

**Affiliations:** ^1^ VTT Technical Research Centre of Finland Ltd., Espoo, Finland; ^2^ Environmental Research and Innovation Department, Luxembourg Institute of Science and Technology, Hautcharage, Luxembourg

**Keywords:** plant cell suspension cultures, dedifferentiation, bioprocess optimization, bioreactors, plant secondary metabolites (PSMs)

The cultivation of plants and plant cells *in vitro* is a landmark in the history of plant science. Tissue and cell suspension culture techniques are not only important for basic research, but also for crop improvement, the preservation of endangered species, and the production of high added-value compounds. Interest in the use of plants and plant cells as “factories” has recently been re-ignited following a slump in publications after the prolific 1990s (Thorpe, 2012). This reflects the immense potential of plant cell cultures coupled with advances in bioprocess engineering (Werner et al., 2018).

Plant cell suspension cultures can be established when explants are induced to re-enter the division cycle and proliferate under the stimulus of exogenously supplied phytohormones. The resulting amorphous mass of undifferentiated cells, known as a *callus*, can be converted into a suspension culture of individual cells by agitation in liquid medium.

Plant cells can be cultivated in bioreactors ranging in capacity from a few liters up to several cubic meters. The standardized processes are season-independent, devoid of pathogens and contaminants, and compliant with current good manufacturing practices (Georgiev et al., 2018). Although the cultivation of microbes for the production of industrially-relevant compounds via precision fermentation is well understood (Hilgendorf et al., 2024), less is known about the cultivation of plant cells in bioreactors. This is due to challenges such as shear sensitivity, slower growth compared to microorganisms, aggregate formation, foaming, and the maintenance of critical process parameters such as oxygen transfer rates at the pilot and industrial scales. Some of these aspects are addressed in this Research Topic of *Frontiers in Plant Science*.

Verdú-Navarro et al. discuss different types of bioreactors for the cultivation of plant cells. Continuous stirred-tank reactors (CSTRs) and wave reactors are prominent in research laboratories and industrial processes because single-use and autoclavable versions are available. Glass CSTRs (allowing light to reach the cells) and stainless-steel ones (which maintain darkness) rely on impellers to ensure uniform nutrient distribution and gas exchange. However, mechanical agitation can harm cells that are particularly shear sensitive. As an alternative, 2D rocking bioreactors simulate waves, thus achieving aeration and nutrient distribution by swaying cells back and forth in sealed bags containing the culture medium.

The use of plant cells to produce recombinant proteins by transient expression or stable transformation has garnered interest in the pharmaceutical industry because plant cells offer advantages in terms of cost, quality and safety compared to traditional microbial and animal cell platforms (Schillberg et al., 2013). Navarre et al. explore the importance of transformation procedures by studying the efficiency of two expression cassettes encoding a viral envelope glycoprotein (gP) when introduced into *Agrobacterium tumefaciens* and delivered to tobacco BY-2 cells. They found that a replicative DNA transposon was integrated into the T-DNA of *Agrobacterium* strain LBA4404 when the gP coding sequence was under the control of the cauliflower mosaic virus 35S promoter, but not when a plant-specific promoter was used instead. They also showed that the GV3101 strain did not trigger any plasmid alterations. For these reasons, the authors recommend the use of controls at each step of the transformation procedure to prevent any deleterious effects.

Ideally, a plant cell line should be tested in different configurations before establishing the most cost-effective bioprocess. Although this can be expensive, such a strategy is particularly useful with rarer species for which little information is available, and this aspect is explored in two articles in this Research Topic.

Titova et al. show that the careful characterization of cell lines is important before proceeding with further bioprocess development and upscaling for commercial production. They used well-established Vietnamese ginseng (*Panax vietnamensis*) cell lines originating from the same rhizome. Six cell lines grown in flasks were compared in terms of specific growth rate, productivity, and maximum biomass. The medium composition had a negligible effect, but a specific inoculum density was influential on a case-by-case basis. A significant finding was that the cell cultures produced a more diverse range of bioactive compounds: ginsenosides, including derivatives that are not produced by the plant itself but are known from other species. The accumulation of ginsenosides correlated with the degree of cell aggregation. The authors conclude that the cell culture method can be a feasible alternative to the conventional cultivation of ginseng plants, which is time-consuming and environmentally harmful, enabling the sustainable production of ginsenosides for use in functional foods, cosmetics, and healthcare products.

Raikar et al. explore the use of *in vitro* cell suspension cultures derived from feijoa or pineapple guava (*Acca sellowiana*) floral buds to produce secondary metabolites. Various parameters affecting the process were investigated, such as callus induction from different regions of floral buds, biomass accumulation, and the synthesis of bioactive compounds. The optimal inoculum density, hormone concentrations and sugar sources were identified for initiating and growing feijoa cell cultures. Additionally, the study examined the effects of methyl jasmonate (MeJA) on the accumulation of secondary metabolites, increasing the production of phenolic compounds. One of these metabolites (arctigenin, which is known for its anti-tumor, anti-inflammatory and anti-colitis effects) was identified in feijoa floral buds and cell cultures, suggesting the potential for industrial-scale production. Future research should explore alternative strategies with safer plant growth regulators and focus on the identification of elicitors that further enhance arctigenin production.

Hairy root cultures are another well-known plant-based production system, but one that uses differentiated rather than undifferentiated cells. Alcalde et al. report the proteomic analysis of *Centella asiatica* hairy and adventitious roots and the use of multivariate statistics to identify and quantify proteins, thus increasing our understanding of the effects of *rol* genes on plant metabolism. The hairy roots were divided into three categories based on their ability to produce centelloside, and a significant correlation was found with certain protein biomarkers. The study quantified the copy number and expression levels of *rol* genes in the transgenic lines and found that *rolD* was strongly expressed in the lines producing the highest levels of centelloside. This also correlated with the biomarker ornithine cyclodeaminase (OCD), an enzyme involved in proline synthesis and root growth. The study suggests that OCD could enhance stress tolerance and growth regulation in hairy roots, making it a useful tool for the assessment of plant biofactories. The work also shows that the insertion site, rather than the number of transgene copies, has a more pronounced influence on elevated expression levels and subsequent protein translation.

## Author contributions

SH: Writing – original draft, Writing – review & editing. SL: Writing – original draft, Writing – review & editing. HR: Writing – original draft, Writing – review & editing. JR: Writing – original draft, Writing – review & editing. GG: Writing – original draft, Writing – review & editing.
